# Apis-Mellifica

**Published:** 1895-04

**Authors:** 


### HOMŒOPATHIC PHYSICIAN,

A MONTHLY JOURNAL OF

HOMŒOPATHIC MATERIA MEDICA AND CLINICAL MEDICINE.

"If our school ever gives up the strict inductive method of Hahnemann, we are lost, and deserve only to be mentioned as a caricature in the history of medicine."—CONSTANTINE HERING.

Vol. XV. APRIL, 1895. No. 4.

### EDITORIAL.

APIS-MELLIFICA.—Continuing the notes upon this remedy from the March number we find that Apis has headache accompanied by diarrhœa. The headache is relieved as the diarrhœa increases. The headache is worse in a warm room and from reading. This is similar to Pulsatilla. The brain feels tired, and head feels too large. The patient cannot concentrate his thoughts. The headache is on the left side and in the left eye. It may be in the whole left side of the head with redness and swelling of the left cheek with nausea and vomiting. Sometimes the pain is on the right side of the head when it extends to the eye, which must be kept closed. The headache continues from ten A. M. to six P. M.

There is numbness and tingling in the brain, which may extend down left arm and left leg.

Apis is a great remedy in hydrocephalus, the delirium being marked by *sudden shrill screams.* This screaming is the great characteristic of the Apis delirium. There is also a variety of spasmodic actions, among which may be mentioned boring of the head deep into the pillows; squinting; grinding of the teeth; twitching of one side of the body and paralysis of the other side. These symptoms can be found in Lippe's

*Materia Medica,* if the reader is fortunate enough to have a copy.

Dr. Lippe added the following notes: Spasm of the toes. The big toe is turned up, is immovable, and very painful. There is great tension of the scalp. It feels as if drawn tightly over the head. There is inability to hold up the head. Sudden convulsions followed by unconsciousness and fever. Stramonium has sudden screaming at night like an Indian.

To these notes the editor makes the following additions in reference to the screaming:

Sudden thrilling cries; frequent screaming spells; screaming out during sleep, are all various forms of the keynote of Apis, which is *sudden screams.*

Screaming spell in an infant every day at five o'clock, so hard as to cause scrotal hernia, Calcarea-carbonica.

Sudden screaming from earache, Aurum-metal.

Sudden screaming of infants, Anacardium, Carbo-veg., Hyos.

Screaming loudly as if calling some one, Anacardium.

With reference to the boring of the head into the pillows the following comparisons of the editor are offered:

Boring of the head into the pillows, Apis, Arnica, Belladonna, Croton-tiglium, Hellebore, Stramonium.

Rolling of the head, Cina, Hyoscyamus.

Rolling of the head night and day with moaning, Hellebore.

Desire to lie down to roll the head from side to side, Hellebore.

Cannot keep the head still, Ferrum.

Convulsive movements of the head from before backward, Nux-moschata.

Involuntary throwing of the head from side to side until he falls to sleep, Causticum.

Rolling of the head from side to side, Zinc.

Throwing head from side to side with continual crying, Colchicum (Dr. M. Preston).

Rolling of head from side to side during sleep. Sleep with eyes half open. Moaning, Lyc.

Throwing head backward from shocks in the brain, Lycopodium.

Weight in the back of the head. Tendency of the head to incline backward against the back of the chair, probably from congestion of the cerebellum, Ignatia.

Referring to the tension of the scalp, we offer the following comparisons:

Sensation as if the skin of the head were contracted, Argentum-nitricum, Arnica, Cocc-c.

Stiffness of the muscles of the face with throbbing headache, Agaricus.

Sensation as if the skin of the face were drawn tight, Cannabis-indica.

Skin of the face feels as if white of egg had dried upon it, Phosphoric acid.

Skull feels as if stretched, Sambucus.

The eye symptoms are very characteristic. Inflammation of the eye, with intense photophobia and great lachrymation; the eyelids being highly œdematous, are symptoms that can be found in any materia medica. The comments by Dr. Lippe were: Pulsatilla has sensation of mucus hanging over the eye like a veil. The patient tries in vain to wipe it away. Apis has the sensation of mucus in the eye in much the same way. Arsenicum, the eyes are very dry. Apis has violent shooting pains in the inflamed eye. Apis has rheumatic inflammation of the left eye extending over the cheek, like smooth erysipelas, and with scalding tears flowing over it. The sclerotica is intensely red, with opacity of cornea. It looks smoky. The conjunctiva on the eyelids looks like raw meat, and the edges of the eyelids are agglutinated.

Apis has œdematous swelling of the face. There is œdematous swelling of the eyelids. The face has a waxy look. This reminded the lecturer that Phosphorus has swelling of the face beneath the eyes, and that Kali-carbonicum has swelling over the upper eyelid beneath the eyebrow. This last symptom, by the way, is Dr. Guernsey's keynote for Kali-carbonicum.

Apis has erysipelas of the face, which begins on the lower lip as a blister, and thence spreads over the whole lower jaw, locking the jaw.

The editor can add to the above note that when erysipelas begins on the right side of the face, in the region of the right eye, and spreads over the face to the left Lycopodium is almost certainly the remedy. It would be hard to tell just what number of cases of erysipelas beginning in this manner have fallen under the notice of the editor, all of which were brilliantly cured by Lycopodium.

All the foregoing notes and comments it must be understood, were directly derived from Dr. Lippe's lectures, excepting only such as are directly given by the editor. These latter were collected as before stated during years of study of the materia medica. They are not exhaustive and they do not pretend to be. They are selected because they are prominent indications of prominent remedies.

What has been given could be readily found by any close student of materia medica working industriously and intelligently. The mass of information so acquired would make a formidable equipment for the faithful prescriber in his battle with disease.

It is to be borne in mind that there will be nothing gained by merely reading over this article and then laying the number aside. It is only valuable in proportion as the reader makes it his own by memorizing, or copies it off where it will continually fall under his eye. The best way of doing this would be to get a book well indexed—the *Index Rœrum* is very good—and, taking each individual symptom, put it under that letter of the alphabet which corresponds to its principal word. Thus would a gradual accumulation of valuable symptoms be acquired, forming a convenient manuscript repertory. This method was long ago pointed out by Dr. Yingling in an article entitled "Private Repertories," published in THE HOMŒOPATHIC PHYSICIAN for March, 1891, page 124. It is the method used by the editor, and he has made an accumulation which he hopes some day to publish.

